# Everybody's Page

**Published:** 1889-09-07

**Authors:** 


### Everybody's Page.

Camphor is offensive to mice, and will keep them away from places where it is scattered about.

Twenty cases of smallpox in Sydney scared a Premier into erecting a smallpox hospital which is now a convalescent home, wherein 200 patients are nicely accommodated.

Arrangements are being made for further lectures at the Sanitary Institute. There are no subjects more studied at present among all classes of medical men than those of hygienic science and sanitation. They are as much a portion of a medical man's outfit nowadays as a lancet case or the inevitable stethoscope.

Wealth does not necessarily favour long life. In Professor Humphrey's "Report on Aged Persons," containing an account of 824 individuals of both sexes, and between the ages of 80 and 100, it is stated that 48 per cent. were poor, 42 per cent. were in comfortable circumstances, and only 10 per cent. were described as being in affluent circumstances.

Holidays should be spent out of doors. A holiday spent in attending lectures or exhibitions is neither relaxation nor rest. "The air is a cordial of incredible value," and fresh air is the one cure for the weariness and depression which afflicts town dwellers in the nineteenth century. Before the winter comes with its short day and frequent storms, let us get out of doors as much as possible.

The electric light has proved to be not merely a more brilliant and steady illuminant than any other that can be cheaply produced, but cooler, better for the eyes, and in all respects more healthful. It throws off neither poisonous fumes nor dirt. Two years' use of it in the Post Office Central Savings Bank in London remarkably diminished absences of the staff from illness.

Australians fancy they are less liable to insanity than other people. In 1881, when the last census was taken in New South Wales, the proportion of insane per 1,000 among persons of British nationality was 8·03; among foreigners, 6·87; while among Australians it was only 1·22 per 1,000, owing partly to the fact that fully one-third of the Australian born were children, and insanity was mainly a disease of middle life and old age.

Huxley, in an address delivered in 1881, said of pharmacology: "There can surely be no ground for doubting that, sooner or later, the pharmacologist will supply the physician with the means of affecting, in any desired sense, the functions of any physiological element of the body. It will in short become possible to introduce into the economy a molecular mechanism which, like a very cunningly devised torpedo, shall find its way to some particular group of living elements and cause an explosion among them, leaving the rest untouched."

Dr. J. W. Barrett lately read a paper on the nature of vision in animals, which was the result of experiments made with a view to ascertain with what degree of accuracy the mammalia can see, the investigation having been begun in London in conjunction with Mr. Lang. The eyes of rabbits, guinea pigs, horses, cats, dogs, mice, cows, and monkeys had been examined, and it was found that with the exception of monkeys those animals did not possess the power of accommodation, and that their vision was decidedly inferior to human sight. The monkey, however, possesses some power of accommodation.

"How do you feel this morning, grandmamma?" "I don't know, child. The doctor has not come yet."

Eye Hospitals are on the increase; twenty large towns which have general hospitals have also eye hospitals.

Before 1860, only about half-a-dozen different kinds of microbes had been discovered. There are now more than 200 varieties with which science is more or less familiar.

The world has been said to be peopled with labourers and loafers, but Bishop Bathurst now gives a new division—benefactors and malefactors. Not to be a malefactor you must take pains to benefit your fellow-men; a good creed this for our poverty-stricken charities.

When Indian shawls were more popular than they are now, it was popularly supposed that real ones could be detected from imitation French make by the smell, as the former were always scented with patchouli. A certain M. Piesse, however, imported the plant, and did a thriving business in Indianising shawls for a long time before the manœuvre was discovered.

Dr. Neild, of Melbourne, is more in favour of health and sanitary societies than of large hospitals. He recently said that if one-tenth part of the effort which was made to keep up and extend hospital accommodation were employed in propagating the knowledge of hygienic necessities and showing people how much better it was to keep themselves healthy, the results would be much more satisfactory than they were at present.

Amongst the specimens of children's essays lately published was a very pathetic one on "The Doctor" by a small boy, who eventually died of brain fever: "The doctor has seen me three times for the purpose, cuz I have headaches. My mother looks at me, and cries when he's gone. I never tells mother I have headaches, except it hurts me very much. I love my mother. I wish my head was same as other boyses. Yesday I arskt Webster if he ever felt dizzy, and he said no. All boys I ask says no. What the doctor gives me makes feel me worser. But mother likes me to take it, so I don't mind. I wish I was a man, but I'd rather be a woman like mother. Doctors hav'nt niced houses. There is bottles all round and no washin. Doctors hav'nt loud voices like men you hears in the street, but their eyes are brighter. I am not so frightened of doctors as of perlice."

Anthrax is one of the most deadly and rapidly fatal diseases known. It has been for ages a terrible scourge amongst cattle, horses, sheep, and many other animals all over the world. And it is conveyed to man. Sheep-shearers and those who handle wool are liable to it. In them it is known as "wool-sorters' disease." Microbes in the blood of animals dead from anthrax had been discovered as early as the year 1844, but much importance had not been attached to them; people did not know what to make of the fact. In 1863 Davaine, guided by the result of Pasteur's researches, declared he had proved them to be the cause of the disease. By introducing a drop of blood from a sheep just dead of the disease into other sheep, they took anthrax immediately and died. Pasteur investigated the matter with his unerring skill, and declared that Davaine was right. It is proper to say that at this period Koch, a young unknown German physician, had taken the matter in hand, and proved, with a skill almost equal to Pasteur's, that the microbe of anthrax was the cause of the di ease.

